# Biotransformation of β‐hydroxypyruvate and glycolaldehyde to l‐erythrulose by *Pichia pastoris* strain GS115 overexpressing native transketolase

**DOI:** 10.1002/btpr.2577

**Published:** 2017-11-20

**Authors:** Yu‐Chia Wei, Stephanie Braun‐Galleani, Maria José Henríquez, Sahan Bandara, Darren Nesbeth

**Affiliations:** ^1^ Dept. of Biochemical Engineering University College London, Bernard Katz Building London WC1E 6BT U.K.; ^2^ School of Medicine, Conway Institute, University College Dublin, Dublin 4 Ireland

**Keywords:** *Pichia pastoris*, transketolase, whole cell biocatalyst, l‐erythrulose, product inhibition

## Abstract

Transketolase is a proven biocatalytic tool for asymmetric carbon‐carbon bond formation, both as a purified enzyme and within bacterial whole‐cell biocatalysts. The performance of Pichia pastoris as a host for transketolase whole‐cell biocatalysis was investigated using a transketolase‐overexpressing strain to catalyze formation of l‐erythrulose from β‐hydroxypyruvic acid and glycolaldehyde substrates. Pichia pastoris transketolase coding sequence from the locus PAS_chr1‐4_0150 was subcloned downstream of the methanol‐inducible AOX1 promoter in a plasmid for transformation of strain GS115, generating strain TK150. Whole and disrupted TK150 cells from shake flasks achieved 62% and 65% conversion, respectively, under optimal pH and methanol induction conditions. In a 300 μL reaction, TK150 samples from a 1L fed‐batch fermentation achieved a maximum l‐erythrulose space time yield (STY) of 46.58 g L^−1^ h^−1^, specific activity of 155 U $g_{\text{CDW}}^{-1}$, product yield on substrate (Y_p/s_) of 0.52 mol mol^−1^ and product yield on catalyst (Y_p/x_) of 2.23g $g_{\text{CDW}}^{-1}$. We have successfully exploited the rapid growth and high biomass characteristics of Pichia pastoris in whole cell biocatalysis. At high cell density, the engineered TK150 Pichia pastoris strain tolerated high concentrations of substrate and product to achieve high STY of the chiral sugar l‐erythrulose. © 2017 The Authors Biotechnology Progress published by Wiley Periodicals, Inc. on behalf of American Institute of Chemical Engineers *Biotechnol. Prog.*, 34:99–106, 2018

## Introduction

Small molecule pharmaceuticals, comprising one or more chiral centers, remain a major product type in modern medicine, comprising two of the top five selling drugs globally in 2016, and five of the top 15.^1^ Optically pure synthesis of small molecule pharmaceuticals by conventional chemi‐synthetic routes tends to require multiple steps, each of which is typically defined by low product yield. As such, complex small molecules with multiple chiral centers tend to have high production costs due to the need for multiple chemi‐synthetic steps.

This challenge has led to the development of alternate “toolbox” platforms^2^, ^3^ including biological routes to controlled, stepwise assembly of the chemical species required to synthesize a final, desired molecule. Such biological routes typically involve the application of enzymes in the place of chemical catalysis, for “biocatalysis” to produce the final compound, over one or more steps. One advantage of enzymes is their promiscuity with respect to the molecules they engage with as substrates, which can enable a given enzyme to be used for a broad range of chemistries.^4^ Enzymes can also provide exquisite regio‐, chemo‐, and stereo‐selectivity for the synthetic steps they catalyze.

In comparison to chemi‐catalytic conditions, which can require extremes of pH and temperature, biocatalysis is typically performed in mild conditions; 25°C‐37°C and pH 7. Mild conditions can be cheaper to maintain than the harsher conditions of chemi‐catalysis. However, harsh conditions are often unavoidable for upstream process steps such as the conditioning of feedstocks to make them physically and biologically compatible with downstream steps. Transitioning from harsh to mild conditions between process steps typically requires further energy or material inputs and brings significant cost implications.^5^ Purified enzymes are often active in only mild conditions, but variants can be sourced or engineered that are stable to extremes.^6^ However, even robust enzyme variants have no capacity to regenerate themselves, as they inevitably accumulate damage and denaturation over time. One option to make enzymes both self‐renewing and tolerant to non‐mild conditions is to deploy them while they are still sequestered within a host cell, so‐called whole cell biocatalysis.

*Escherichia coli* (*E. coli*) remains a popular option for whole cell biocatalysis, due to its genetic tractability, robustness, and rapid growth. However, *E. coli* is vulnerable to lysis by bacteriophage, which are naturally ubiquitous in the environment^7^ and therefore also in agricultural feed streams. *E. coli* cells also undergo increasingly lethal cell stress from below pH 5.5^8^ and above pH 8.^9^ A broad variety of alternative host cells have thus been characterized for their performance as whole cell biocatalysts.^10^ Yeasts are a major alternative, with no pathogens equivalent to bacteriophage and a broader range of pH tolerance, from pH 3 to pH 7.^11^

In addition to robustifying a biocatalytic step, whole cell biocatalysis can also enable assembly of multiple enzymes within a single component, the cell, to achieve complex, multi‐stage synthesis.^12^ These pathway engineering approaches mean that biological routes are emerging as credible alternatives to chemi‐synthesis for production of an ever‐increasing number of small molecule product classes, from biofuels to pharmaceuticals. Engineered pathways can be “hosted” by a cell, or steps taken to integrate some or all of the pathway into the native metabolic networks of the host cell. In the former approach typically lower value, achiral compounds are fed to the host cell and converted to higher value chemicals with defined chiral centers.^13^ In the latter approach, often termed metabolic engineering, the cells need only be cultivated using a cheap carbon source, often crude waste streams from agriculture, and they will in effect convert this material to a product of higher value, such as a biofuel.^14^

A particular class of enzymes known as transketolases (EC 2.2.1.1) have been investigated as biocatalytic tools due to their ability to form the asymmetric carbon‐carbon bonds commonly required for chemical synthesis of a broad range of pharmaceuticals, agrochemicals, materials, and food ingredients.^15^ In nature transketolases require thiamine diphosphate as a cofactor and provide a regulatory function in cellular metabolism, linking the pentose phosphate pathway to glycolysis by catalyzing transfer of a two‐carbon fragment from xylulose to ribose sugars, forming heptulose sugars. Biotechnologists have shown that this activity can be exploited to drive an irreversible reaction when providing β‐hydroxypyruvate (HPA) as the donor of the two‐carbon fragment, resulting in carbon dioxide as one of the reaction products. Transketolases have typically been sourced from genomic data mining of yeasts^16^ and bacteria^17^ and used for production of a number of different products including food flavorings, and synthetic precursors of chiral amino alcohols which are valuable intermediates in synthesis of small molecular therapeutics.^18^

The stereospecificity and enantioselectivity typical of transketolases often results in bio‐catalyzed reaction routes that are more atom‐efficient than their chemo‐catalytic counterparts for a given synthesis step.^19^ Key measures used to assess the efficacy of a given transketolase system for biocatalysis are activity, substrate‐specificity, and inhibition by products or substrates. If necessary to achieve sufficient conversion, the cost of enzyme purification can also be a key performance measure. *E. coli* transketolase has been reported to be effective in production of l‐erythrulose when in solution,^20^ immobilized within a microreactor^17^ and within living *E. coli* cells.^21^

The methylotrophic budding yeast, *Pichia pastoris* (*P. pastoris*), is an established laboratory‐scale recombinant protein production platform^22^ and is gaining traction as an industrial‐scale platform for production of enzymes^23^ and therapeutics.^24^ Characteristically low specific yield does not prevent high volumetric yields of recombinant protein^25^ as *P. pastoris* can grow rapidly to high biomass concentration, with 40% v/v WCW typically achieved. Methylotrophic yeasts have also been observed to tolerate extreme overexpression of the alcohol oxygenase 1 (Aox1p) enzyme essential for methanol utilization, to a level where 30% of cell biomass can be taken up with this enzyme during growth phase.^26^

The effectiveness of *P. pastoris* as a whole cell biocatalyst has been investigated for ester biosynthesis,^27^ acetophenone reduction.^28^ d‐Amino acid oxidation^29^ and kinetic resolution of racemic α‐methylbenzylamine to 99% ee (*R*)‐α‐methylbenzylamine.^13^ A cell‐surface display strategy has also been used for expression of active lipase by a *P. pastoris* whole cell biocatalyst.^30^

Previous work showed that overexpression of a native transketolase within an *E. coli* whole cell biocatalyst could achieve asymmetrization of the prochiral compounds β‐hydroxypyruvate (HPA) and glycolaldehyde (GA) to generate up to 20 mM l‐erythrulose (ERY), for subsequent steps in a *de novo* biocatalytic pathway, over a 17.5 h reaction period.^21^ In this study, we sought to establish if the same asymmetrization could be achieved using *P. pastoris* cells overexpressing a native transketolase. We also wished to test if the higher biomass achievable in *P. pastoris* could be harnessed to achieve higher levels of ERY production compared to the *E. coli* whole cell biocatalyst.

## Materials and Methods

### Construction of the TK150 native transketolase overexpressor strain

All DNA manipulations, unless otherwise stated, were performed using standard molecular biology techniques. The genome of *P. pastoris* strain GS115 (Invitrogen, Carlsbad, CA), recently assigned^31^ as *Komagataella phaffii* (ATCC 20864), encodes a transketolase (TK) open reading frame (ORF) at locus PAS_chr1–4_0150 (Genbank: CAY67980.1). A single colony of *P. pastoris* strain GS115 was boiled in water to provide genomic DNA template for preparative polymerase chain reaction (PCR). Primers CACAATGTCTGATCTCTTAGC (start codon underlined) and AAACTACGCATGAACAGACTC (stop codon underlined) were provided by Eurofins MWG Operon (Ebersberg, Germany) and used to PCR‐amplify a 2.04 kb fragment from the PAS_chr1–4_0150 locus^32^ encoding the TK ORF. The resultant PCR fragment was subcloned into the pCR‐BluntII‐TOPO vector (Invitrogen) between a pair of Eco RI restriction sites and its identity confirmed by sequencing. The TK ORF fragment was then subcloned from pCR‐BluntII‐TOPO into the *P. pastoris* expression vector, pAO815 (Invitrogen), by non‐directional ligation into a lone Eco RI site and a Hind III digest used to confirm orientation. The resultant plasmid, designated pAOX0150‐TK, was linearized with the restriction enzyme Stu I and used to transform *P. pastoris* GS115 following manufacturer's instruction.^33^ Successful transformation generated a new strain, designated TK150, in which the methanol utilization phenotype was preserved as Mut+ and the histidine biosynthesis phenotype was converted from His4 to HIS4.

### Shake flask cultivation

Cells were typically cultivated in 50 mL buffered glycerol complex medium (BMGY) broth (1% w/v yeast extract, 2% w/v peptone, 100 mM potassium phosphate pH 6, 1.34% w/v yeast nitrogen without amino acids, 1% v/v glycerol, and 0.4 µg/mL biotin) in a 250 mL shake flask incubated at 30°C, with 250 rpm agitation for 15 h, after which an OD_600_ = 2 was reached. Glycerol stocks for cell cryopreservation were made by pelleting cells at OD_600_ = 2 by centrifugation at 13,000 rpm (Eppendorf 5415R centrifuge, Eppendorf UK, Ltd, Cambridge, UK) for 5 min at room temperature and resuspending cells in a solution of 5 volumes of BMGY plus 3 volumes of 80% glycerol, then transferring 1.5 mL aliquots into 2 mL cryo‐vials. Working cell banks (WCB) of 20–30 vials were generated for each strain. Typically, 100 mL of BMGY medium in a 250 mL conical flask were inoculated with 1 mL of a glycerol stock. For investigation of recombinant TK activity, cells were grown to OD_600_ = 2 then pelleted as before. To induce expression of TK under control of the AOX1 promoter, cells were resuspended again to OD_600_ = 2 in buffered methanol (BMMY) complex medium (recipe as BMGY but 1% v/v glycerol substituted for 0.5% v/v methanol) and incubated as above with pure methanol added to 0.5% v/v every 24 h post‐induction initiation.

### Bioreactor cultivation

A 100 mL culture of TK150 cells in BMGY media in 500 mL shake flasks was incubated at 30°C with agitation at 250 rpm until OD_600_ = 80 was achieved. 40 mL of this culture were used to inoculate 600 mL of fermentation basal salts medium (BSM) containing 4% w/v glycerol, to give an initial OD_600_ of 5 in a 1L Infors bioreactor (Multifors 1, INFORS HT, Switzerland). BSM consisted of 26.7 mL 85% w/v H_2_PO_4_, 0.93 g CaSO_4_, 18.2 g K_2_SO_4_, 14.9 g MgSO_4_.7H_2_O, 4.13 g KOH, 40 g glycerol, and 12 mL “Pichia Trace Metal 1” (PTM1) solution (6.0 g/L CuSO_4_5H_2_O, 0.08 g/L Nal, 3.0 g/L MnSO_4_.H_2_O, 0.2 g/L Na_2_MoO_4_.2H_2_O, 0.02 g/L H_3_BO_3_, 0.5 g/L CoCl_2_, 20.0 g/L ZnCl_2_, 65.0 g/L FeSO_4_.7H_2_O, 0.2 g/L biotin, and 5.0 mL/L 96% H_2_SO_4_) per liter dH_2_O. During this initial trophophase of glycerol batch growth, a dissolved oxygen set point of 20% was used for control of pure oxygen feeding. Bioreactor cultivation was performed as per Invitrogen protocol.^29^ Three rates of methanol feed were introduced for induction, 3.6 mL/h/L initially (M1), then 7.3 mL/h/L (M2), and a final feed rate of 10.9 mL/h/L (M3).

### Preparation of solutions of whole and disrupted cells for biocatalysis

Bioreactor and shake flask samples were centrifuged at 13,000 rpm for 20 min at 4°C to pellet cells. Whole cells were re‐suspended in pH 7 Tris buffer and placed on ice to inhibit further growth. A Soniprep 150 sonicator (MSE, London, UK) was used to subject samples to a 10 s cycle of 100% amplitude sonication, followed by 10 s rest, three times.

### Reaction conditions

A 100 μL solution containing whole cells or disrupted cells was initially incubated with 15 μL of a 2.4 mM solution of the cofactor thiamine pyrophosphate and 15 μL of 9 mM MgCl_2_ at room temperature with constant shaking at 150 rpm for 20 min. 50 μL of hydroxypyruvate and 50 μL of glycolaldehyde, both at 300 mM, were then added in addition to 70 μL of 50 mM Tris buffer to a final reaction volume of 300 μL and substrate concentration of 50 mM. Higher substrates concentrations, of up to 2 M, were achieved by using the required volumes of 7 M stock solutions of each substrate, again in a total final reaction volume of 300 μL.

### l‐Erythrulose measurement

The Dionex HPLC system (Camberley, UK) and its monitored software Chromeleon client 6.60 were used for reaction analysis. The system contains an AD20 UV/vis absorbance detector, a FAMOS autosampler, an oven and a GP50 gradient pump. A 15 min isocratic assay, as reported by Mitra and Woodley,^34^ was used to analyze HPA and ERY, using a 300 mm × 7.8 mm HPX‐87H Reverse Phase Column (Bio‐Rad Amines, CA, USA). Mobile phase was prepared with 0.1% trifluoroacetic acid (TFA) and dH_2_O at a constant temperature of 60°C, flow rate 0.6 mL/min. Prior to analysis samples were quenched and diluted into 10% in 0.1%v/v TFA.

## Results and Discussion

### Growth and methanol tolerance of TK150 strain in shake flasks

**Figure 1:**
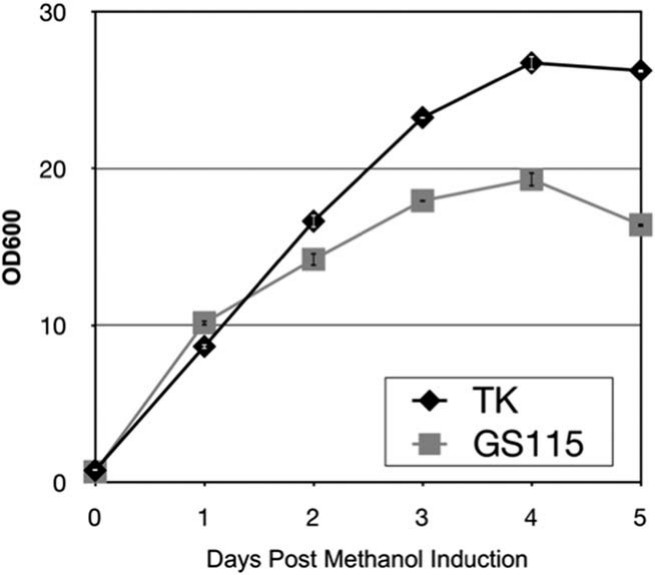
Shake flask growth performance of *P. pastoris* TK150 transketolase overexpressor strain. *P. pastoris* strains GS115 or TK150 (TK) were cultivated in 250 mL shake flasks in 50 mL BMGY media. Samples were removed at the indicated time points and OD_600_ measured. Error bars indicate standard deviation among three biological repeats of the experiment.

**Figure 2:**
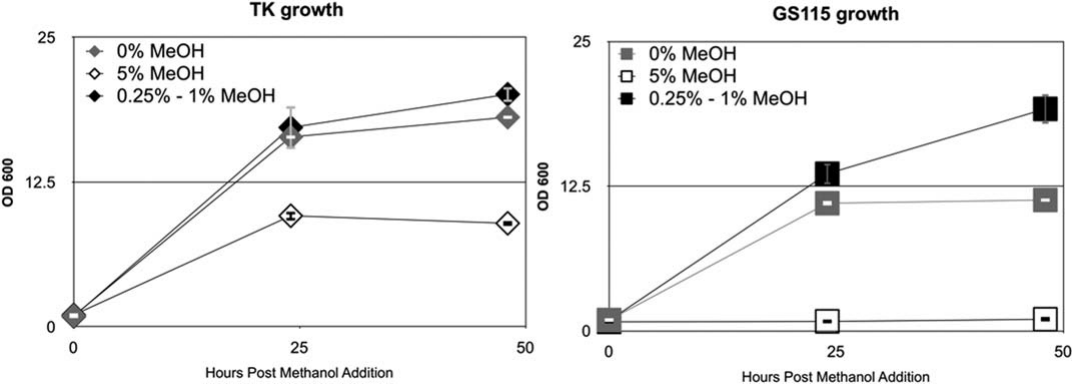
Methanol tolerance of the *P. pastoris* TK150 transketolase overexpressor strain. *P. pastoris* strains GS115 or TK150 (TK) were cultivated in 250 mL shake flasks in 50 mL BMMY media supplemented with methanol (MeOH) to the percentage v/v indicated. Samples were removed at the indicated time points and OD_600_ measured. Error bars indicate standard deviation among three biological repeats of the experiment.

A novel *P. pastoris* GS115 strain, designated “TK150,” was generated in which a native transketolase, encoded at the locus CAY67980.1 (Genbank), was placed under the control of the AOX1 promoter. Performance in shake flasks achieved by the TK150 strain was comparable to that of the parental strain in the presence of methanol (0.5% v/v) but the TK150 strain achieved higher optical density (OD) from day three post‐induction onward (Figure 1). Nocon et al.^35^ showed that transketolase overexpression increased metabolic flux in *P. pastoris* X‐33 strain so we hypothesized transketolase overexpression may also increase the capacity of *P. pastoris* to metabolize methanol. To test this hypothesis, we cultivated TK150 and GS115 strains in 200 mL shake flasks in 50 mL complex media (see Methods) supplemented with methanol in concentrations of 0%, 0.25‐1%, and 5% v/v. Figure 2 shows that both TK150 and parental GS115 strains grew to a similar level in the presence of 0.25‐1% v/v methanol. The highest concentration of 5% v/v methanol suppressed completely growth of the parental GS115 strain but still enabled growth of TK150, decreasing growth rate by half when compared to the supplementation of 0.25‐1% v/v. Interestingly, the TK150 strain also reached higher cell density than GS115 in the methanol‐free condition.

### Transketolase activity in whole and disrupted TK150 cells

**Figure 3:**
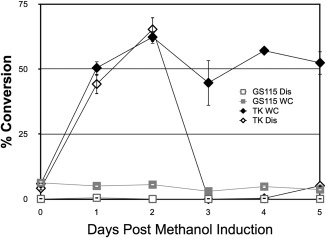
Transketolase activity of TK50 strain cultivated in shake flasks. *P. pastoris* strains GS115 or TK150 (TK) were cultivated in 0.5% v/v methanol BMMY media. At the indicated time points samples were removed and prepared for incubation with 50 mM β‐hydroxypyruvate and 50 mM glycolaldehyde either as whole (WC) or disrupted cells (Dis), as described in Methods. The concentration of l‐erythrulose produced was measured by HPLC and plotted as a percentage of the starting concentration of substrates (50 mM), as a level of conversion. Error bars indicate standard deviation among three biological repeats of the experiment.

We next established the profile of methanol‐induced transketolase activity appearance in whole cells and cells disrupted by sonication (Figure 3). Activity was measured by percentage conversion of 50 mM HPA and GA substrates to ERY. TK150 strain cells were grown in the absence of methanol to OD_600_ = 2 and from then onwards cultivated in the presence of daily batches of methanol to 0.5% v/v. Figure 3 shows that, for the TK150 strain, transketolase activity peaked at 48 h post‐induction, as measured in both whole cells and sonicates. For TK150 whole cells, conversion remained within a 45–60% range for from 1 to 5 days post‐induction. This robustness in performance for whole cells may be due to cellular homeostatic processes, such as chaperone activity, working to maintain the folding and stability of cellular proteins during shake cultivation and incubation with fed substrates. However, for disrupted TK150 cells conversion dropped dramatically at day three, down to less than 10%. This steep drop might be due to the fact little or no homeostatic activity is present in disrupted cells to maintain protein stability during incubation with fed substrates. No significant activity was observed for the parental GS115 strain in the same experiments.

### Transketolase activity across pH 5–9

**Figure 4:**
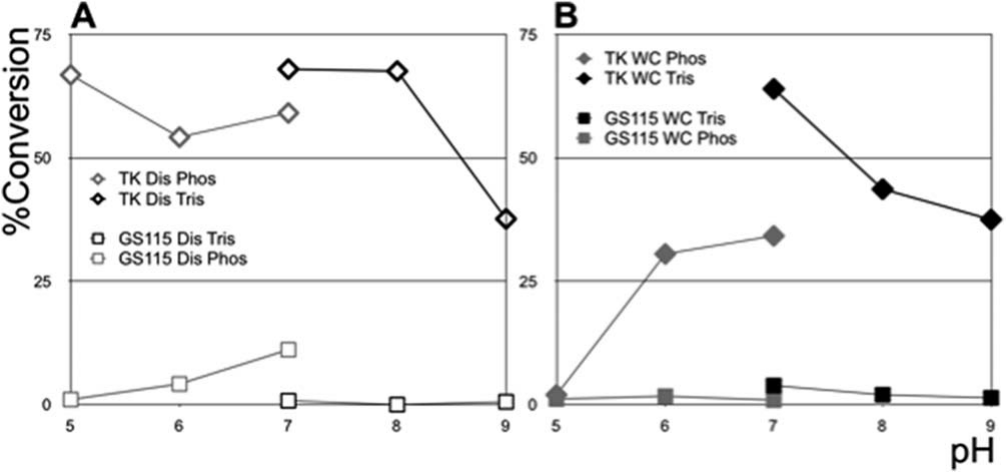
Influence of pH and buffer choice on transketolase activity. *P. pastoris* strains GS115 or TK150 (TK) were cultivated in 0.5% v/v methanol BMMY media as in Figure 3. Samples were removed and prepared for incubation with 50 mM β‐hydroxypyruvate and 50 mM glycolaldehyde either as whole (WC) or disrupted cells (Dis). During this preparation samples were resuspended in either Tris (Tris) or Phosphate (Phos) buffer at the indicated final pH. Conversion of substrates to l‐erythrulose was measured and plotted as in Figure 3. Data set depicted is representative of three biological repeats of the experiment.

Conditioning steps, such as adding solvents or altering pH, are sometimes necessary to render a given feed stream compatible with the addition of enzymes or whole cell biocatalysts in subsequent steps of a bioprocess. As such, robustness to a range of pH values can be an industrially advantageous property for biocatalysts. To investigate pH tolerance, we used whole cells and sonicates from 48‐h induction in shake flasks to determine optimal pH conditions for bioconversion (Figure 4). We measured percentage conversion of 50 mM HPA and GA to ERY in the presence of buffers at a range of different pH values as indicated in Figure 4. Across pH 5–7, cell sonicates maintained their level of activity in a phosphate buffer (Figure 4A). Using a Tris buffer increased sonicate transketolase activity at pH 7 compared to phosphate buffer. This high activity in Tris‐based buffer was maintained at pH 8 but decreased at pH 9.

Counter to our expectations, transketolase activity in TK150 whole cells was much lower in phosphate buffer across pH 5–7 (Figure 4B) compared to sonicates (Figure 4A). Furthermore, whole cell transketolase activity in phosphate buffer at pH 5 was completely abolished (0% conversion) compared to the same condition for sonicates (70% conversion). This observation was unexpected as Chiruvolu et al.^36^ report significant *P. pastoris* cell growth at pH 5. Sauer et al.^37^ measured an intracellular pH of 7.1 within *P. pastoris* cells immersed in pH 5 growth media. Achieving this intracellular pH control requires the employment of cellular energy and mechanisms to drive reverse chemi‐osmosis across cell membranes. Such homeostatic processes may compromise expression of the transketolase transgene, the stability of the enzyme or the permeability of cell membranes to the fed transketolase substrates, via as yet uncharacterized mechanisms. Further research in future will be required to establish if such affects cause the abolished activity at pH 5. The parental GS115 strain resulted in no significant transketolase activity across the different pH conditions (Figure 4).

### Cultivation of TK150 strain to high cell density in a bioreactor

**Figure 5:**
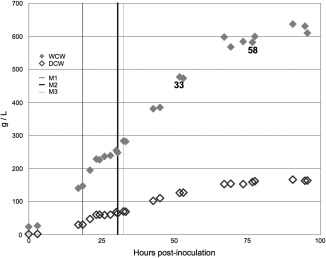
Bioreactor cultivation of TK150 strain to high cell density. Strain TK150 was grown in a bioreactor to high cell density using a protocol in which methanol feeding commenced at the point post‐inoculation indicated by the M1 bar. Methanol feed rate is stepped up at M2 and subsequently at M3. Wet cell weight (WCW) and dry cell weight (DCW) for each sample are indicated (see inset key). Samples taken at zero, 33 and 58 h after the start of induction (M1) were used to test biocatalytic performance. Data set depicted is representative of three biological repeats of the experiment.

A major advantage of *P. pastoris* for industrial application is its ability to achieve higher levels of biomass, of up to 60% WCW/v, than the typical values obtained for *E. coli* or *Saccharomyces cerevisiae* (*S. cerevisiae*). This can result in high levels of a recombinant enzyme or protein per unit volume even if per cell yield is low.^38^ Consequently, we cultivated *P. pastoris* strain TK150 to high cell density and measured the level of bioconversion achieved. Figure 5 details the typical growth profile observed during cultivation of *P. pastoris* cells in a 1 L Infors bioreactor, reaching wet cell weight (WCW) of 600 g/L using the cultivation regime reported by Templar et al.^39^ Samples were taken immediately prior to the start of methanol feeding (indicated as M1 in Figure 5) and then 33 h and 58 h after the start of methanol feeding.

### Bioconversion by the TK150 strain at high cell density

**Figure 6:**
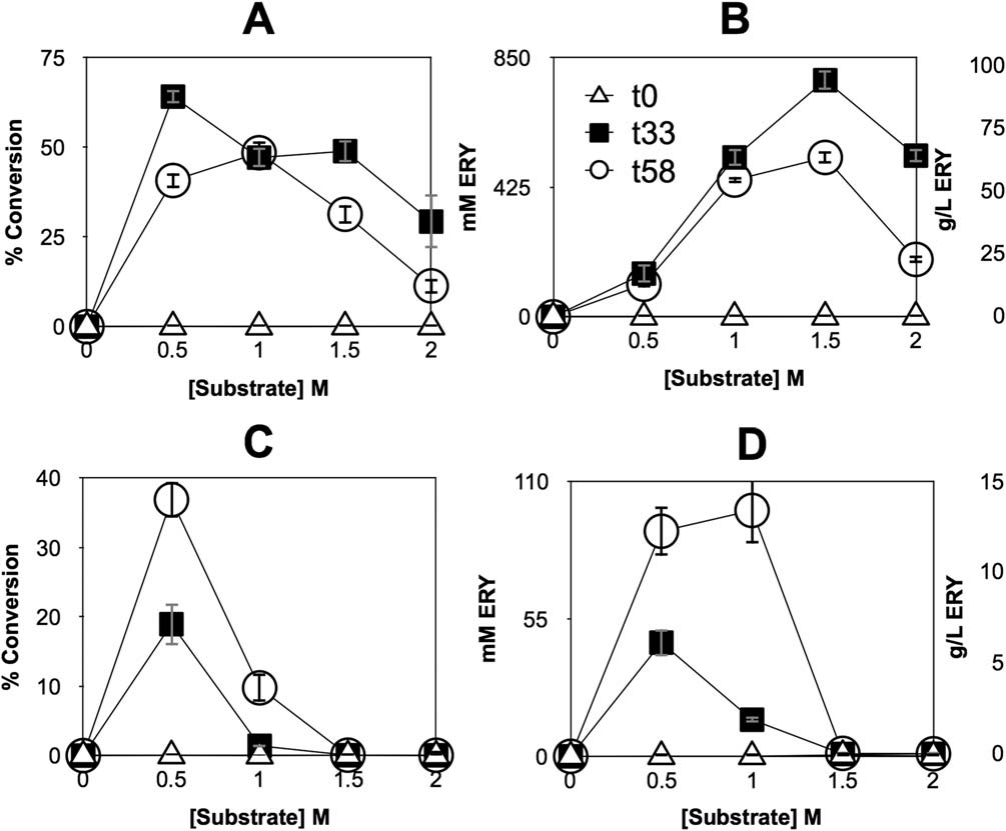
Effect of substrate concentration, cell density and induction period on TK150 strain whole cell biotransformation. Bioreactor samples, taken at zero, 33 and 58 h post induction, were used to test biocatalytic performance for solutions of whole cells (A and B) and cell sonicates (C and D). Percentage conversion of substrates to l‐erythrulose (A and C) was plotted alongside accumulation of l‐erythrulose in molar and mass concentration terms (B and D). Error bars indicate standard deviation among three biological repeats of the experiment. Key in plot B applies to all plots.

**Figure 7:**
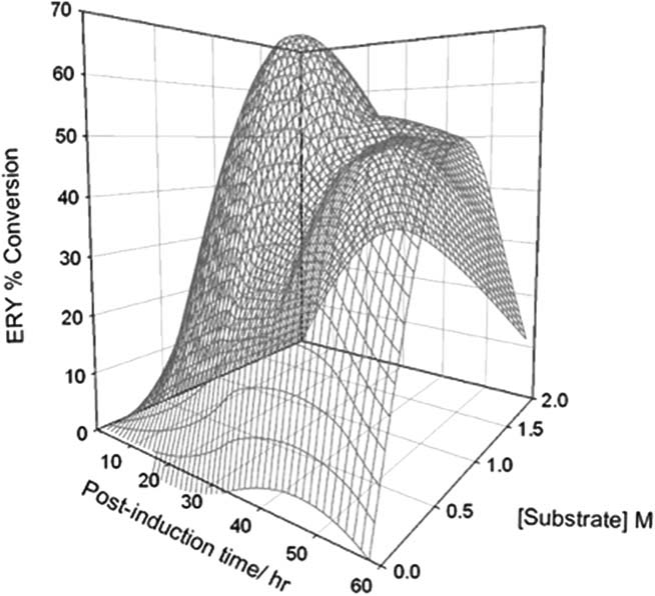
Response surface for TK150 strain whole cell biotransformation. Figure 6E data gathered for percentage conversion of substrates to l‐erythrulose (ERY) were plotted as a function of both the time post‐induction and the initial substrate concentration present in the reaction mixture. Average values were used to generate the response surface. Standard deviation among three biological repeats of the experiment is omitted for graphical brevity.

To establish if the high biomass achievable by *P. pastoris* can translate into enhanced biocatalytic performance, samples taken from the Infors 1 L bioreactor at zero, 33 and 58 h post‐induction were assessed for their volumetric activity in bioconversion of HPA and GA to ERY (Figure 6). For whole cells, the greatest conversion percentage, nearing 65%, was achieved with the 33‐h sample and with 0.5 M substrates (Figure 6A). The highest amount of ERY produced was 776.4 mM, again with the 33‐h post induction sample but with a concentration of 1.5 M substrate provided (Figure 6B). For sonicates, conversion fell relative to whole cells, with the highest bioconversion level, of 38%, achieved with the 58‐h post‐induction sample and 0.5 M substrates (Figure 6C). The highest concentration of ERY produced by sonicates was 110 mM, again with the 58‐h post‐induction sample but with 1.0 M substrates (Figure 6D). A response surface (Figure 7) was generated to map regions of high whole cell bioconversion to ERY as a function of induction period and substrate concentration. For cells harvested at 33 h post methanol induction, maximum conversion was achieved upon feeding with 0.5 M substrates. For cells harvested at 58 h post methanol induction, maximum conversion was achieved upon feeding with 1 M substrates. These distinct maxima were not expected. In general, the percentage of viable cells decreases over the idiophase of high cell density bioreactor cultivation. We suggest this viability difference between the 33 h and 55 h post induction samples is likely to be a key determinant of the different performance profiles. Future research will be needed to clarify this.

### TK150 strain whole cell biocatalyst performance

Research into the usefulness of *P. pastoris* as whole cell biocatalysts continues to accumulate, including the use of killed^40^ or permeabilized^41^, ^42^ whole cells. Schrewe et al.^10^ discuss a selection of performance metrics, distinct from those commonly applied for cell‐free enzymes, to meaningfully characterize and compare whole cell biocatalysts. Table 1 shows a selection of Schrewe metrics for the biocatalytic performance of the whole cells in this study, for the sample taken 33 h post‐induction. Ingram et al.^21^ constructed an *E. coli* whole cell biocatalyst overexpressing native transketolase for conversion of β‐hydroxypyruvic acid and glycolaldehyde to l‐erythrulose. They reported a maximum l‐erythrulose production of 20 mM after a 17.5 h reaction duration. This equates to a STY of 13.7 mg L^−1^ h^−1^ which is 3,400‐fold lower than the activity of 46.58 g L^−1^ h^−1^ we report here for *P. pastoris* whole cell biocatalysis.

**Table 1:** Schrewe Metrics Whole Cell Biocatalysis. Date Gathered Using the 33‐h Post Induction Sample of *P. pastoris* Strain TK150 Cultivated in a 1 L Bioreactor

Reaction Values	
Starting [GA], [HPA]	1.5 M
[ERY] after 2 h reaction	0.7764 M
µM ERY per min	6469.8
[ERY] after 2 h reaction	93.2 g/L
g/L DCW in sample	125.25
g/L DCW in reaction	41.75
*Schrewe metrics*	
STY (*g*_product_ L^−1^ h^−1^)	46.58 g L^−1^ h^−1^
Specific activity (U $g_{\text{CDW}}^{-1}$) (*U* = μmole min^−1^)	155 U $g_{\text{CDW}}^{-1}$
*Y*_p/s_ (mol_product_ ${\text{mol}}_{\text{substrate}}^{-1}$)	0.52 mol mol^−1^
*Y*_p/x_ (*g*_product_ $g_{\text{CDW}}^{-1}$)	2.23 g $g_{\text{CDW}}^{-1}$

## Conclusions

The property of *P. pastoris* that already significantly benefits its use in recombinant protein production, namely rapid growth to high biomass, was also used here to intensify biocatalysis. A robust and novel recombinant *P. pastoris* whole cell biocatalyst, TK150, overexpressing a native transketolase gene was developed as a hosted, one‐step biocatalytic pathway to bring about asymmetric carbon‐carbon bind formation in the production of l‐erythrulose from achiral substrates. The *P. pastoris* whole cell biocatalyst remained active from pH 7–9, and achieved performance metrics in line with other *P. pastoris*‐based biocatalysts and orders of magnitude greater than a bacterial equivalent.
